# Disentangling leaf-microbiome interactions in *Arabidopsis thaliana* by network mapping

**DOI:** 10.3389/fpls.2022.996121

**Published:** 2022-10-06

**Authors:** Kaihang Li, Kexin Cheng, Haochen Wang, Qi Zhang, Yan Yang, Yi Jin, Xiaoqing He, Rongling Wu

**Affiliations:** ^1^College of Biological Sciences and Technology, Beijing Forestry University, Beijing, China; ^2^National Engineering Research Center of Tree Breeding and Ecological Restoration, Beijing Forestry University, Beijing, China; ^3^Departments of Public Health Sciences and Statistics, Center for Statistical Genetics, The Pennsylvania State University, Hershey, PA, United States

**Keywords:** *Arabidopsis thaliana*, leaf-microbiome interactions, network mapping, path analysis, microbial networks

## Abstract

The leaf microbiota plays a key role in plant development, but a detailed mechanism of microbe-plant relationships remains elusive. Many genome-wide association studies (GWAS) have begun to map leaf microbes, but few have systematically characterized the genetics of how microbes act and interact. Previously, we integrated behavioral ecology and game theory to define four types of microbial interactions – mutualism, antagonism, aggression, and altruism, in a microbial community assembly. Here, we apply network mapping to identify specific plant genes that mediate the topological architecture of microbial networks. Analyzing leaf microbiome data from an *Arabidopsis* GWAS, we identify several heritable hub microbes for leaf microbial communities and detect 140–728 SNPs (Single nucleotide polymorphisms) responsible for emergent properties of microbial network. We reconstruct Bayesian genetic networks from which to identify 22–43 hub genes found to code molecular pathways related to leaf growth, abiotic stress responses, disease resistance and nutrition uptake. A further path analysis visualizes how genetic variants of *Arabidopsis* affect its fecundity through the internal workings of the leaf microbiome. We find that microbial networks and their genetic control vary along spatiotemporal gradients. Our study provides a new avenue to reveal the “endophenotype” role of microbial networks in linking genotype to end-point phenotypes in plants. Our integrative theory model provides a powerful tool to understand the mechanistic basis of structural-functional relationships within the leaf microbiome and supports the need for future research on plant breeding and synthetic microbial consortia with a specific function.

## Introduction

Plants, as the main producer, are colonized by a variety of microorganisms that form complex microbiomes, including bacteria, fungi, archaea and protists (Flues et al., 2018; Hassani et al., 2018). These microorganisms interact dynamically with plants and influence their hosts’ growth and development. Plant microbiome can enhance plants growth directly or indirectly through increasing abiotic stresses tolerance (Rolli et al., 2015; Poudel et al., 2021; Trivedi et al., 2022), nutrient acquisition (Van Der Heijden et al., 2015; Wang et al., 2021), disease resistance (Mendes et al., 2011; Ritpitakphong et al., 2016), and pathogen inhibition *via* the synthesis and excretion of antibiotics (Hernandez-Salmeron et al., 2016). For example，when plants face biotic and abiotic stresses，those stresses trigger host signals to recruit beneficial microbes (the“cry for help”hypothesis) in the rhizosphere. The beneficial bacteria recruited can directly help the plant resist stress (Wang and Song, 2022).Some hub microorganisms play an important role in maintaining the stability of the microbiome and can indirectly affect plant health (Brachi et al., 2022). The composition and functioning of the microbiomes even can predict plant health (Wei et al., 2019) and help mitigate the negative consequences of climate change (Jansson and Hofmockel, 2020). Microbiomes play a pivotal role in plant growth and health, but the genetic factors involved in microbiome assembly are still in their infancy (Escudero-Martinez et al., 2022; Oyserman et al., 2022).

Most studies on plant microbiomes have focused on rhizosphere microbial communities and their functioning rather than on those of phyllosphere. The phyllosphere microbiomes may play essential but often overlooked roles in nutrient acquisition, abiotic stress tolerance, and disease suppression of plants (Gao et al., 2021; Li et al., 2022). Experimental studies have demonstrated that the phyllosphere harbors diverse microbial communities that influenced ecosystem functioning (Laforest-Lapointe et al., 2017; Chen et al., 2020; Liu et al., 2020; Christensen et al., 2021; Massoni et al., 2021; Xu et al., 2022). Recent studies stress the importance of understanding the mechanisms underlying how plant-microbe interactions in the phyllosphere could influence host survival and fitness in the context of global change (Perreault and Laforest-Lapointe, 2022; Zhu et al., 2022). Several studies have illustrated that host genotypes can influence the composition of the leaf microbiome (Bodenhausen et al., 2014; Wagner et al., 2016; Brachi et al., 2022), but little is clear how the plant shapes its leaf microbiota and how the leaf microbiome contributes to plant phenotypic traits (Gupta et al., 2021; Hawkes et al., 2021; Pfeilmeier et al., 2021).

It has been recognized that microbial interactions affect plant traits, plant evolution (Rodriguez et al., 2019; Delaux and Schornack, 2021) and ecosystem function (Getzke et al., 2019). Despite tremendous efforts to reveal the molecular mechanisms of microbial interactions (Xiong et al., 2017; Saleem et al., 2019), their role in modulating plant function through the context of ecological networks has been little studied. This may be due to the fact that the microbiota is a highly-packed ecosystem, making it extremely difficult to discern and quantify individual microbial interactions. It has been found that the in phyllosphere of *Arabidopsis* plays an important role in maintaining the growth of *Arabidopsis*, and dysbiosis of phyllosphere does cause plant disease (Chen et al., 2020). Therefore, as a model plant, understanding the interaction mechanism between *Arabidopsis* and microorganisms will provide a theoretical basis for subsequent studies. By integrating behavioral ecology and game theory, Wu and team developed mathematical descriptors for quantifying and characterizing different types of microbial interactions, including mutualism (two microbes promotes each other), antagonism (two microbes inhibit each other), aggression (a stronger microbe is aggressive to a weaker microbe), and altruism (one microbe benefit the other), in ecological communities at any large scale (Jiang et al., 2019; Wang et al., 2019). These mathematical descriptors, named Wu’s descriptors for convenient mention, have been biologically validated by designing and conducting a series of cultural experiments using the fish and bacteria, respectively (Jiang et al., 2019, 2021). Based on Wu’s descriptors, Wu et al. (2021) have further formulated conceptual hypotheses on the strategic choice of organisms’ behavior in complex communities, including the golden threshold hypothesis, the competition-to-cooperation shift hypothesis, the Fibonacci retracement mark hypothesis and the surrender-resistance hypothesis. He et al. (2021) introduced Wu’s descriptors into a GWAS setting, proposing a network mapping tool to study the genetic architecture of microbial networks.

The main goals of the paper are to (1) Quantify networks of microbe interactions on *A. thaliana* leaves; (2) Identify microbe hubs and estimate their heritability; (3) Identify plant variants associated with the microbe networks; and (4) Link host genotype to phenotype/fitness through their microbiome. Here, we implement network mapping to dissect the internal workings of the leaf microbiome for *Arabidopsis*, using the published GWAS data from a well-designed multi-trial and multi-year experiment (Brachi et al., 2022). Key plant genetic variants that influenced leaf hub microbes responsible for *A. thaliana* fitness were identified. Through our reanalysis, we attempt to illustrate a more comprehensive picture of the genetic architecture underlying the leaf microbiome of *A. thaliana*. First, network mapping allows us to reconstruct four ecologically different types of microbial networks based on Wu’s descriptors. Thus, we can map host genes for the topological architecture of microbe-microbe interactions and interdependence. Second, it has been increasingly clear that microbial traits are not only determined by key host QTLs (Quantitative trait loci), but also through their epistatic networks (He et al., 2021). Network mapping can draw the host genetic landscape of microbial interactions. Taken together, network mapping can discern both hub microbes in the leaf microbiome and hub QTLs in the genetic architecture of the host and characterize the impact of these different types of hubs on plant fitness. We further implement path analysis to chart the roadmap of genotype–phenotype linking through microbial interactions as the endophenotypes.

## Results

### Ecological networks of the leaf microbiota

**Figure 1:**
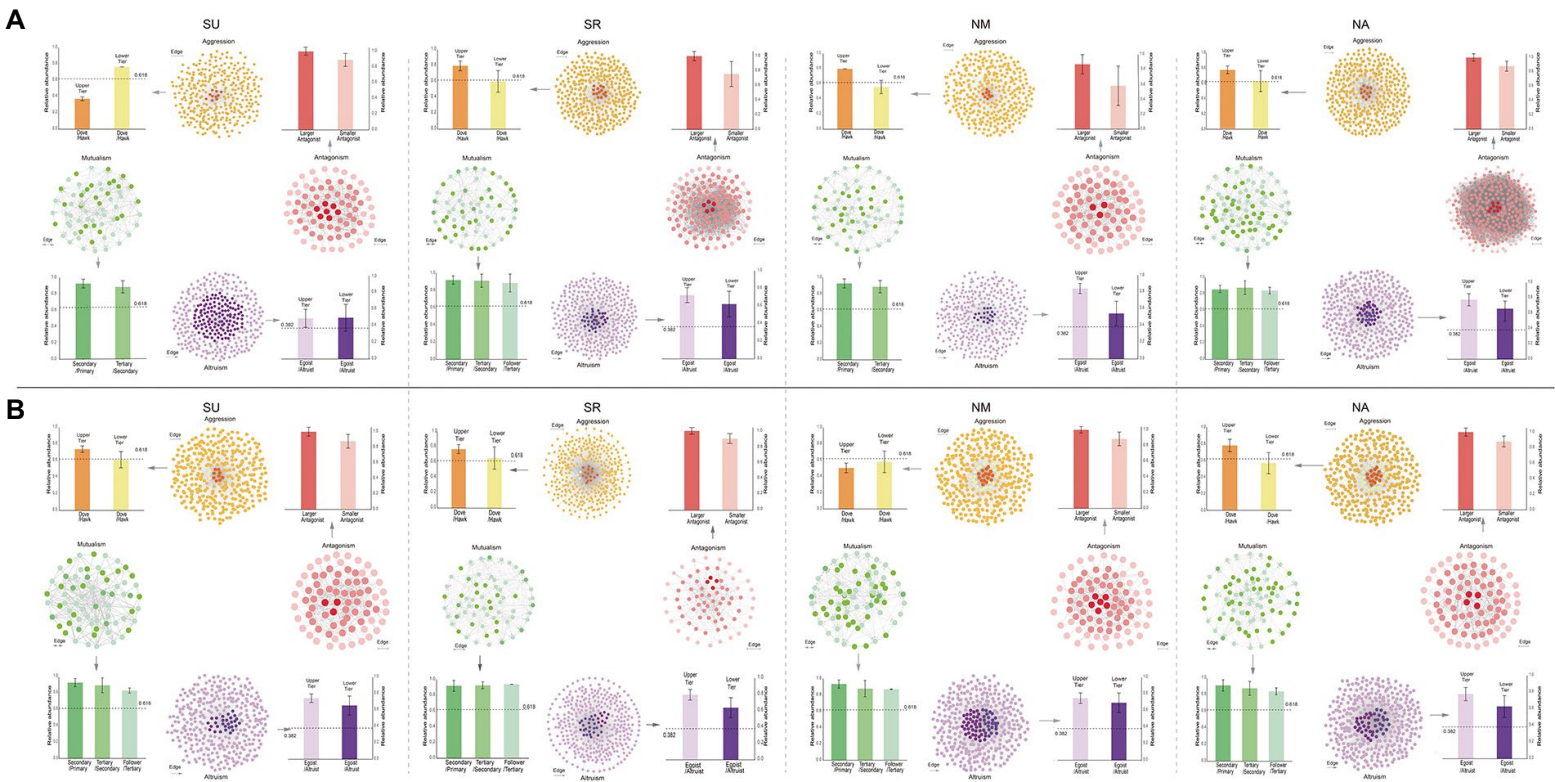
Social networks of microbiome in the leaf of *Arabidopsis thaliana*. **(A)** The results of four experiments in 2012, **(B)** The results of four experiments in 2013. In each plot, left: Mutualism network constructed by doubly-arrowed edges representing microbes cooperation; right: Antagonism network composed of doubly-T-shaped edges denoting the mutual conflict of the microbes; top: Aggression network represented by singly-T-shaped edges specifying how a microbe (as a hawk) aggresses upon others (doves; color metrics indicate three hierarchies of aggression); bottom: Altruism network characterized by singly-arrowed edges which illustrates how a microbe (altruist) benefits other microbes (egoists) at its own expense (color metrics indicate three hierarchies of altruism).

Organisms in communities interact in various ways to form complex behavioral relationships as they strive to acquire resources for survival and reproduction. In order to quantify the networks of microbe interactions on *A. thaliana* leaves, we calculated the relative OTU (Operational taxonomic units) abundance of different members in mutualism, antagonism, aggression and altruism interaction networks (See Materials and methods). Figure 1 illustrates the four networks reconstructed with Wu’s descriptors for the leaf microbiome of *Arabidopsis* at four different sites in 2 years (eight experiments). In the mutualism network, we calculate the relative abundances of the secondary leaders over the primary leaders, the tertiary leaders over the secondary leaders and the secondary leaders over the followers in the eight experiments (in green, Figure 1). All these relative values are obviously larger than 0.618, which complies with the golden threshold hypothesis, stating that a larger microbe tends to cooperate with a smaller microbe when the relative abundance of the latter to the former is beyond 0.618 (Wu et al., 2021). By comparing the abundance of hawks and doves in the aggression network, we find that the ratios of the hawks-doves to the hawks ranged from 0.373 to 0.798 (the upper tier) and the ratios of the doves to the hawks-doves is between 0.557 and 0.765 (the lower tier; in yellow, Figure 1). Many of these ratios are lower than 0.618, which is consistent with the second aspect of the golden threshold hypothesis, stating that a larger microbe tends to exploit a small microbe when the relative abundance of the latter to the former is below 0.618 (Wu et al., 2021). In the altruism network, the relative abundance of egoists over altruists at both the upper and lower tiers are obviously larger than 0.382 (in purple, Figure 1). This is in agreement with the Fibonacci retracement mark hypothesis, stating that for its self-interest, a smaller microbe may “trick” a larger microbe when the relative abundance of the former to the latter is beyond 0.382 (Wu et al., 2021). In the antagonism network, the relative abundance of smaller antagonists over larger antagonists ranges from 0.668 to 0.903 (in red, Figure 1), which is in agreement with the surrender-resistance hypothesis. These hypotheses regarding microbial interactions can unravel the quantitative mechanisms of how microbes cope with others to gain their maximum benefits and, ultimately, affect community assembly structure, organization, and function. It seems that both bacteria and fungi on the *Arabidopsis* leaves empirically obey these hypotheses (Figure 1; Supplementary Table 2).

### Hub microbes and OTU heritability

**Figure 2:**
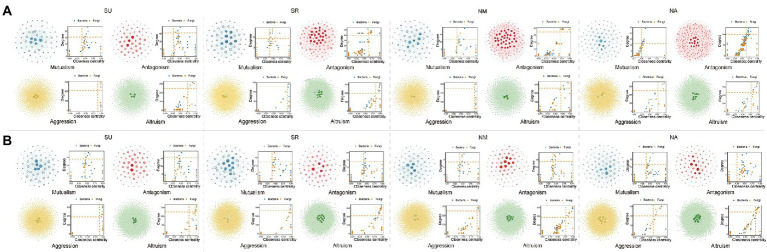
Ecologically hub OTUs of the co-occurrence network. **(A)** The results of four experiments in 2012, **(B)** The results of four experiments in 2013. In each network, hub microbes are highlighted in border colors. The distribution of ‘Hub microbes’ in four different microbial networks was based on degree and closeness centrality values. These two values of each OTU within each network were given at the right. The red dotted line represents the screening cutoffs of ‘Hub microbes’ corresponding to each network. Visualization was done with Gephi for four microbial networks.

**Figure 3:**
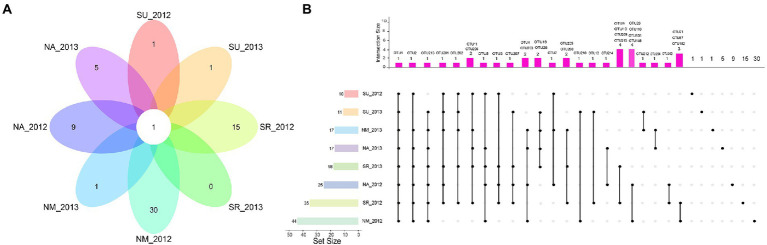
Venn plot for hub OTUs. **(A)** Flower plot shows the number of common and unique hub OTUs in eight experiments, **(B)** UpSet plot for eight experiments. Horizontal bars on the left represent the total number of hub OTUs in each experiment. Deep pink bars represent the number of hub OTUs of each intersection indicated by connected dots.

According to the ecological networks in each environment, we calculated degree-centrality parameters to determine the relative importance of bacteria and fungi in each network and statistically identify the hub taxa by the higher degree and closeness centrality in the four microbial networks. In total we identify 96 hub OTUs in microbial networks from eight experiments, with 57 bacterial hubs and 39 fungal hubs (Figure 2; Supplementary Table 3). These hub microbes are from bacterial phyla: Proteobacteria (46 OTUs), Bacteroidetes (5 OTUs), Actinobacteria (4 OTUs), and Firmicutes (2 OTUs), and fungal phyla: Basidiomycota (19 OTUs), Ascomycota (18 OTUs), and unclassified fungi (2 OTUs). In order to understand the differences of microbial hubs in different environments, we show the results by combining with the Venn diagram and the Upset diagram (Figure 3). The following results can be drawn from the figure that OTU2 (Proteobacteria, *Pseudomonas* sp.) and OTU 213 (Ascomycota, *Tetracladium* sp.) are detected in seven experiments, while fungi OTU 201 (Basidiomycota, *Itersonilia perplexans*) is detected in six experiments. OTU 11 (Proteobacteria, uncultured), OTU 5 (Proteobacteria, *Variovorax* sp.), OTU 202 (Ascomycota, *Tetracladium maxilliforme*) and OTU 206 (Basidiomycota, *Tremellales* sp.) are detected in five experiments. OTU1 (Proteobacteria, *Sphingomonas* sp. TSBY-34) is the only one detected in each site and year (Figure 3).

After calculating the broad-sense heritability (*H*^2^) of the abundance of individual OTUs in eight experiments. We did not find large *H*^2^ for the OTUs, ranging from 0 to 0.2287. Across all eight experiments, 15 hub OTUs are detected to be heritable (with *H*^2^ > 0.10; Supplementary Table 4).

### Hub QTLs associated with the microbial networks

**Figure 4:**
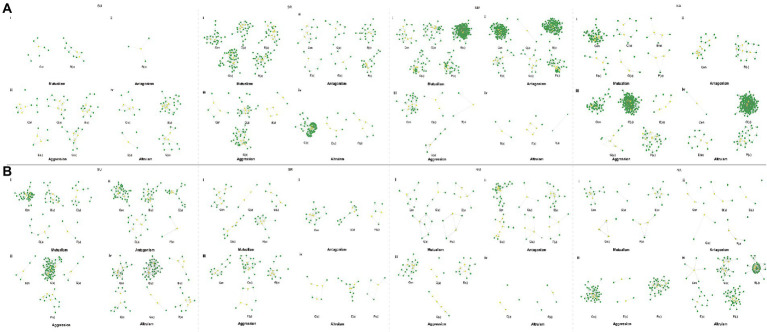
Bayesian networks of the significant SNPs from microbial networks. **(A)** The results of four experiments in 2012, **(B)** The results of four experiments in 2013. Each node reports a SNP and hub QTLs (SNPs) were colored in yellow. The network characteristic indices were described by connectivity (Con), closeness [C(u)], betweenness [B(u)], eccentricity [E(u)], eigencentrality [G(u)], and PageRank [P(u)].

We calculate six network property indices to describe networks’ emergent properties and map significant QTLs for each index in eight experiments (Supplementary Figure 1). The population structure (*Q*) and relative kinship (*K*) are performed by Admixture and EMMAX, respectively, to control spurious associations (Kang et al., 2010). Supplementary Figure 2 illustrates the quantile-quantile plots of the *p* value distributions based on the model without consider *Q* and *K*, the *Q* model, and the *Q* + *K* model. We calculate the genomic inflation factor *λ* of the three models in R (Supplementary Table 5). The model with *λ* close to 1 is chosen to perform association analysis. We also investigate candidate genes within ~10 kb windows on each side of associated SNPs by software PLINK. The genes of *R*^2^ > 0.8 were retained. Finally, we identify 139–727 significant SNPs responsible for microbial network properties in eight experiments (MAF > 0.05; Supplementary Table 6). We find that SNP-based heritability (*h*^2^) for network properties varies from 0% to 12.79% (Supplementary Table 7). Considering epistatic interactions among different genes have been increasingly recognized to play an important role in genetic control, we implemented Bayesian QTL networks to reconstruct genetic networks involving all significant SNPs for each network parameter. In the Bayesian QTL networks (Figure 4; Supplementary Table 8), 28–66 hub QTLs are excavated in eight experiments. There are 40 pleiotropic QTLs including those in the region of *ADA2B (AT4G16420)* and *AT5G02880 (HAL3A)*, detected to influence multiple properties of various microbial networks (Supplementary Table 8). Hub gene *AT1G78070* is identified in two experiments (NM_2012 and SR_2013), while *AT1G23060 (MDP40)* identified in NA_2012 and SU_2013. Gene annotation analysis suggests that a number of hub genes detected are biologically relevant, playing roles in leaf growth, abiotic stress responses, disease resistance, and nutrition uptake (Supplementary Table 8).

Hub gene *ALG10* (*AT5G02410*) encodes alpha1,2-glucosyltransferase (ALG10) that is required for oligosaccharide biosynthesis and subsequently for normal leaf development and abiotic stress response (Farid et al., 2011). The inactivation of ALG10 in *Arabidopsis* results in the activation of the unfolded protein response and increased salt sensitivity. Hub gene *AT5G11250* encodes an atypical TIR-NBS protein (Toll/interleukin-1 receptor- nucleotide-binding site) acting as a regulator of the hormonal response to stress and is required for plant survival and robustness to environmental perturbations (Sarazin et al., 2015). Gene *DDF2* encodes a member of the DREB subfamily A-1 of ERF/AP2 transcription factor family (DDF2), which is expressed in all tissues but most abundantly expressed in rosette leaves and stems. Overexpression of this gene results in the reduction of gibberellic acid biosynthesis and helps the plants increase their tolerance to high-salinity levels. Hub gene *ADA2B (AT4G16420)* encodes a transcriptional co-activator ADA2b in *Arabidopsis* responses to abiotic stress. It is required for the expression of genes involved in abiotic stress either through modulation of histone acetylation in the case of salt stress or affecting nucleosome occupancy in low temperatures response (Vlachonasios et al., 2011).

Hub gene *GLIP2 (AT1G53940)*, encoding a GDSL motif lipase/hydrolase–like protein, plays a role in pathogen defense *via* negative regulation of auxin signaling (Lee et al., 2009). AtLPK1 is a plasma membrane-localized L-type lectin-like protein kinase 1, which is encoded by hub gene *AT4G02410* in this study. Overexpression of AtLPK1 confers the pathogen resistance to infection by *Botrytis cinerea* and regulates salinity response in *Arabidopsis thaliana*, which implicates that AtLPK1 plays essential roles at both abiotic and biotic stress response (Huang et al., 2013). Hub gene *AT1G25550*, encoding nitrate-inducible NIGT1.1/HHO3 proteins, involves in regulating nitrate signaling and phosphorus starvation signals in *Arabidopsis* (Maeda et al., 2018). Hub gene *HAL3A* (*AT3G18030*) expresses HAL3-like protein A which is related to salt and osmotic tolerance and plant growth (Espinosa-Ruiz et al., 1999).

### Microbial networks as endophenotypes for linking host genotype to phenotype

**Figure 5:**
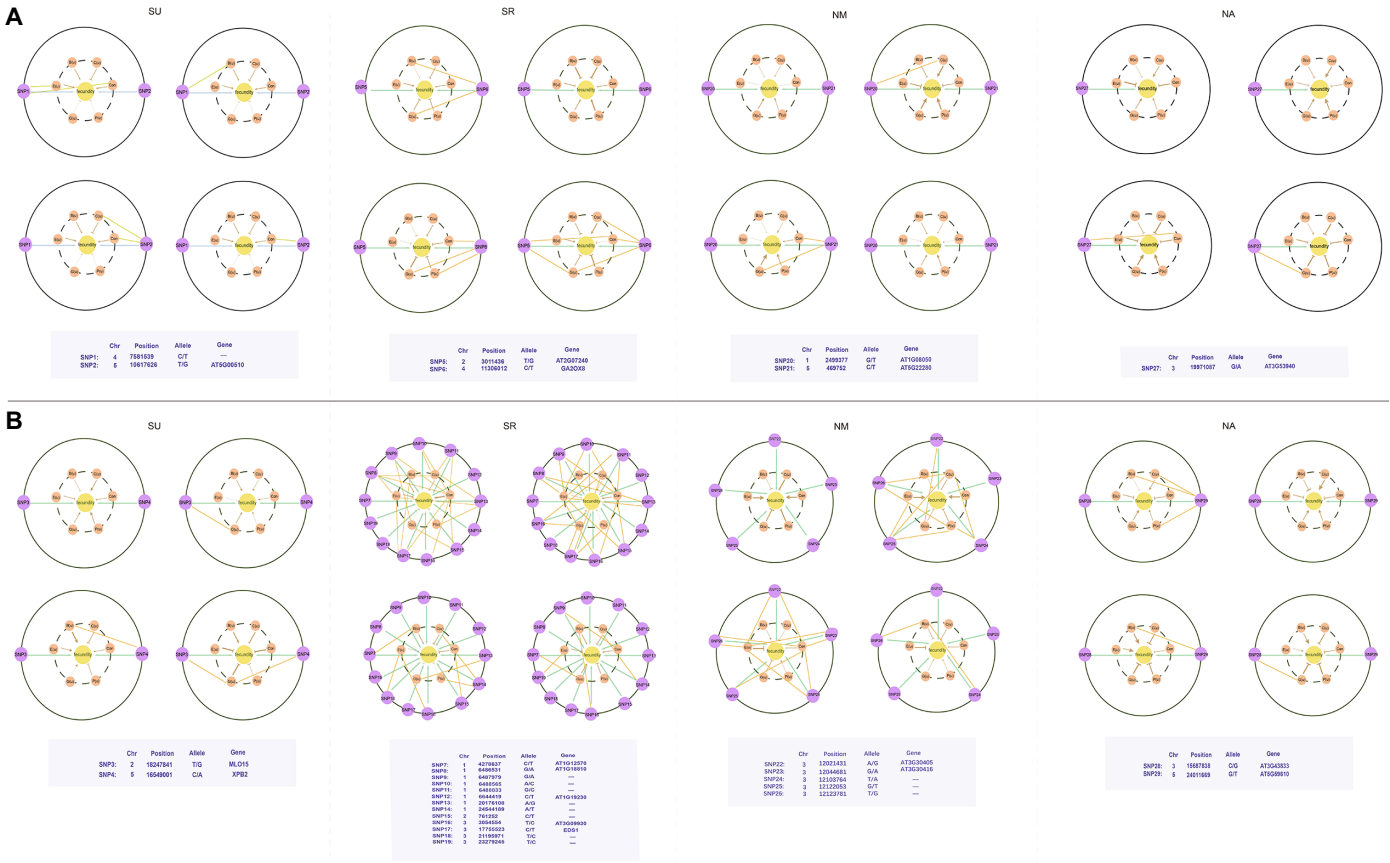
Path analysis revealing how QTLs (outer) affect fecundity as a final phenotype (inner) through microbial networks as an “endophenotype” (middle; described by differences of six emergent property indices). **(A)** The results of four experiments in 2012, **(B)** The results of four experiments in 2013. Path coefficients are denoted by directed lines from SNPs to fecundity (blue) and from networks to fecundity (brown). Arrowed line and T-shaped line represent a positive and negative impact of path, respectively. Correlation coefficients between SNPs and networks are denoted by blue lines. The thickness represents the magnitude of path and correlation coefficients.

We further implement path analysis to dissect the role of microbial networks in linking host genotype (at significant SNPs) to end-point phenotype-fecundity. Although all SNPs display a sizable direct effect on fecundity, they also affect fecundity through the indirect effects of microbial networks as endophenotypes (Figure 5). For example, SNP7, residing in the genomic region of gene *AT1G12570*, is the ortholog of maize IPE1 gene which is involved in pollen exine development. The IPE1 mutant exhibits defective pollen exine and is male sterile (Waadt et al., 2015). *AT1G12570* positively affects fecundity in a direct way, but it also affects fecundity through positive indirect effects of betweenness and eigenvector in the aggression and altruism network in the experiment of SR_2013.

## Discussion

As many studies have been devoted to understanding the diversity of the rhizosphere microbiota, increasing attention has been paid to studying the leaf microbiome, despite its disconnection to soil, often considered to be a site for relatively few microbes (Gong and Xin, 2020). In this study, we apply an advanced network mapping theory for mapping the genetic architecture of microbial interaction networks in the leaf microbiome of *Arabidopsis* (Brachi et al., 2022). From this analysis, we gain new insight into how microbial interactions mediate the leaf microbiome assembly and how plant genes affect plant traits.

The most remarkable feature of this network mapping study is founded on Wu’s descriptors, a group of mathematical equations to characterize and discern different types of ecological interactions, namely, mutualism, antagonism, aggression and altruism, which occur in highly dense microbial community assembly (Jiang et al., 2019, 2021; Wang et al., 2019). Each of these interaction types describes a different aspect of network topology and function, which can better explain how each microbe interacts with every other microbe and how microbial cooperation and competition are reciprocally shifted in response to environmental change (Wu et al., 2021).

Plants are able to regulate a few beneficial microbial taxa to maximize their fitness (Song et al., 2021). Identifying hub taxa is one of the advantages for network analysis (Layeghifard et al., 2017). Hub microbes are highly connected in a microbiome, which are responsible for microbiome structure and maintain the dynamics of the community (Banerjee et al., 2018). Some hub species could act as a mediator to curate a healthier community indirectly even though themselves do not promote plant growth (Bai et al., 2022). Our network mapping using *Arabidopsis* leaf microbiome reveals that phyla Proteobacteria, Actinobacteria, Bacteroidetes, and Firmicutes are dominant groups. Individual strains of Proteobacteria (*Sphingomonas*, *Rhizobium*) and Actinobacteria (*Microbacterium*, *Rhodococcus*) as hub species play an important role in affecting community structure (Carlström et al., 2019). These two phyla are also considered as ecological hub OTUs in this study; especially *Sphingomonas* sp. TSBY-34 serves as a hub species in all 8 experiments. In a recent study, *Sphingomonas* showed significant potential to confer protection to the citrus phyllosphere against pathogen invasion through its iron-competition ability (Li et al., 2022).

We calculate broad sense heritability for individual OTU abundances and identified 15 OTUs that are both heritable and hub microbes in at least one of the eight experiments. In a large-scale longitudinal field study of the maize rhizosphere microbiome, heritable taxa identified were diverse, including 26 Alphaproteobacteria, nine Betaproteobacteria, 12 Actinobacteria, six Verrucomicrobia, and eight Bacteroidetes (Walters et al., 2018). The use of synthetic communities (SynComs) allows for dissecting how one or few community members affect *A. thaliana* and how host genes affect microbiome composition (Bodenhausen et al., 2014; Bai et al., 2015; Durán et al., 2018). Even one single bacterial genus (Variovorax) has an ability to maintain root growth in a complex microbiome (Finkel et al., 2020). A simplified SynComs is able to rescue plant from root rot disease (Li et al., 2021). Heritable hub microbes may serve as the components in the SynComs system in the future research on plant–microbe interactions.

Brachi et al. (2022) identified host genotype effects on the relative abundance of microbial hubs and LSP across sites and years. Their analysis detected a few significant genes for the abundance of heritable hub microbes. Our network mapping characterizes previously undetected QTLs that mediate microbial interactions. There are 40 pleiotropic hub genes, including *ADA2B (AT4G16420)* and *AT5G02880 (HAL3A)*, which are detected to influence multiple types of microbial networks and in eight experiments. Gene annotation suggests that a number of hub genes detected are biologically relevant, playing roles in leaf growth, abiotic stress responses, disease resistance and nutrition uptake. Some of these genes are also found to affect root development (Supplementary Table 8).

Much research has shown that plant genes are a driver to maintain the balance of leaf microbiomes. Plants impaired in genetic networks embrace a dysbiosis leaf microbiome in structure and composition (Liu et al., 2020). The plant genetic network links the leaf microbial community to plant health. For example, a study found that the immunity and cell surface component structuring genes *A. thaliana* mutant harbored less diverse bacterial community in the leaf endosphere relative to the wild type and induced leaf chlorosis (Chen et al., 2020). Our understanding of how plant genotypes impact colonization of specific microorganisms will be instrumental in spurring next-generation plant breeding strategies (Gopal and Gupta, 2016; Kroll et al., 2017).

By integrating network mapping and path analysis, we can characterize how QTLs determine plant phenotype through their direct effects or the indirect effect through leaf microbial networks. We identify these two different paths for SNP-fecundity links. Indirect genetic effects related to these SNPs are mediated through multiple microbial interaction types.

In the near future, modulating the balance of the leaf microbial community by regulating host genetic networks may become a novel approach to improve crop traits and maintain sustainable agricultural development. Our network mapping could hold a great promise to achieve this goal.

## Conclusion

In this study, we quantify the networks of various interaction types for the leaf microbiome in *A. thaliana*. We dissect leaf-microbiome interactions by network mapping to reveal the genetic architecture of microbial interactions and identified hub plant genes, and find that microbial networks and their genetic control vary along spatiotemporal gradients. Even under different circumstances, the interaction between microorganisms is generally consistent with the ecological hypotheses, including the golden threshold hypothesis, the Fibonacci retracement mark hypothesis and the surrender-resistance hypothesis. We conduct path analysis to dissect the roadmap from each of these SNPs to fecundity into the direct path and the indirect paths through microbial network, revealing the “endophenotype” role of microbial networks in linking genotype to end-point phenotypes.

## Materials and methods

### Leaf microbiome experiments

Brachi et al. (2022) performed a GWAS for the leaf microbiome in *A. thaliana*. The study included a panel of 198 *A. thaliana* accessions planted with two replicates in the spring of 2012 and 2013 at four sites located in Ullstorp (lat: 56.067, long: 13.945; SU in short), Ratchkegården (lat: 55.906, long: 14.260; SR in short), Ramsta (lat: 62.85, long 18.193; NM in short), and Ådal (lat: 62.862, long 18.331; NA in short). The bacterial and fungal compositions of each leaf sample was measured by16S rRNA gene and ITS region amplicon sequencing. Accessions were genotyped by a high-throughput sequencing technique, obtaining 186,161 SNPs after quality control. For a detailed description of experimental design, sampling strategy, microbial sequencing, and SNP genotyping, refer to Brachi et al. (2022).

In this study, we choose 200 bacterial OTUs and 200 fungal OTUs at the top-abundance from each site for microbial network inference. These numbers of OTUs account for the top 95.89% of bacterial relative abundance and top 95.50% of fungal relative abundance, respectively, (Supplementary Table 1). OTU1-200 are listed as bacteria and OTU200-400 as fungi. Brachi et al. (2022) measured fecundity for each plant, whose genetic architecture is dissected using both SNPs and microbes.

### Quantify networks of microbe interactions on *Arabidopsis thaliana* leaves

Using Wu’s descriptors, we reconstruct and visualized 400-node interaction networks based on mutualism, antagonism, aggression, and altruism, for 8 year-site experiments. OTU relative abundance is chosen as the trait that determines the strategies of microbial interactions. In the mutualism network, we named hub microbes as primary leaders those whose links are much more than average. The microbes that are directly linked by the primary leaders are called secondary leaders. Those whose routes to the primary leaders are separated by the secondary leaders are regarded as tertiary leaders. The microbes that are linked by the tertiary leaders but not linked by the secondary leaders are called the followers. In the antagonism network, each pair of nodes is linked by two antagonists, i.e., larger antagonists and smaller antagonists. The aggression network is composed of the aggressive group (hawks) and the submissive group (doves). Some microbes may play both hawks and doves if they are aggressive to one member but submissive to another member. The altruism network includes two groups: altruists (that provide benefit to others) and egoists (that receive benefit from altruists).

In order to quantify networks of microbe interactions on *A. thaliana* leaves, we calculated the relative OTU abundance of primary, secondary leaders, tertiary leaders and followers in the mutualism network, the relative OTU abundance of two antagonists in the antagonism network, the relative OTU abundance of hawks and doves in the aggression network, and the relative OTU abundance of altruist and egoists in the altruism network (Wu et al., 2021). Different members were shown by the metric of colors.

### Identify microbe hubs and estimate OTU heritability

The network visualized and hub taxa are identified from each type of microbial network using the Gephi. We calculate the degree of each node in a network. To reduce the bias, we statistically identify the hub taxa with the higher degree and closeness centrality (Gao et al., 2021; He et al., 2021). We also calculate the broad-sense heritability (*H^2^*) of the abundance of individual OTUs in eight experiments using the function *lmer* in *lmer4* R package (Brachi et al., 2022). *H*^2^ was calculated within a year and within an experiment group.

### Emergent properties of microbial networks as phenotype

For each individual, we calculated *Z*mu, *Z*an, *Z*ag, and *Z*al parameters between each pair of genera and used them to reconstruct four corresponding networks, each describing phyllosphere interactions based on a different ecological interaction metric. Six network indices, including connectivity (Con), closeness [C(u)], betweenness [B(u)], eccentricity [E(u)], eigencentrality [G(u)], and Pagerank [P(u)] are calculated among every sample as described previously (Jiang et al., 2021), which are regarded as phenotypic data.

### Identify plant variants associated with The microbe networks

We apply a likelihood approach for detecting significant SNPs that are associated with each of the six network properties for 8 year-site experiments (Wu et al., 2007). We further use *bnlearn* R package to reconstruct Bayesian genetic networks among the significant SNPs detected and plot SNP-SNP interactions. Hub QTLs, playing a key role in genetic networks, are identified.

### Link host genotype to phenotype/fitness through their microbiome

We implement path analysis to test whether a significant SNP affects plant fecundity directly or through an indirect pathway of microbial interactions. We first find the SNPs that affected fecundity through GWAS, then let *g* denotes the genotype, *y* denotes the network property, and *z* denotes the fecundity. We calculated the Pearson correlation between *y* (continuous) and *z* (continuous) across individuals, denoted as *r_yz_*. Let *r_gy_* and *r_gz_* denote the correlations between *g* and *y* and between *g* and *z*, respectively. We calculate the path coefficients *P_z ← g_* from the equation.


$$r_{gz}=P_{z\leftarrow g}+P_{z\leftarrow y}r_{gy},\mathrm{with}\;P_{z\leftarrow y}=r_{yz},$$

where *P_z ← g_* is the direct path from g to *z* and *P*_*z* ← *y*_*r_gy_* is the indirect path through microbial networks.

## Data availability statement

The datasets presented in this study can be found in online repositories. The names of the repository/repositories and accession number(s) can be found in the article/Supplementary material.

## Author contributions

KL analyzed the data and prepared figures and tables. KC analyzed the data and prepared figures and tables. HW analyzed the data and prepared figures and tables. QZ analyzed the data. YY analyzed the data. YJ revised the paper. XH conceived and wrote the paper. RW conceived and revised the paper. All authors contributed to the article and approved the submitted version.

## Funding

This work was supported by Natural Science Foundation of China (31971398).

## Conflict of interest

The authors declare that the research was conducted in the absence of any commercial or financial relationships that could be construed as a potential conflict of interest.

## Publisher’s note

All claims expressed in this article are solely those of the authors and do not necessarily represent those of their affiliated organizations, or those of the publisher, the editors and the reviewers. Any product that may be evaluated in this article, or claim that may be made by its manufacturer, is not guaranteed or endorsed by the publisher.
