# Supplementary figures and images for: Disentangling leaf-microbiome interactions in Arabidopsis thaliana by network mapping
Source: Front Plant Sci. 2022 Oct 6;13:996121. doi: 10.3389/fpls.2022.996121 (PMC9583167; doi:10.3389/fpls.2022.996121)

A

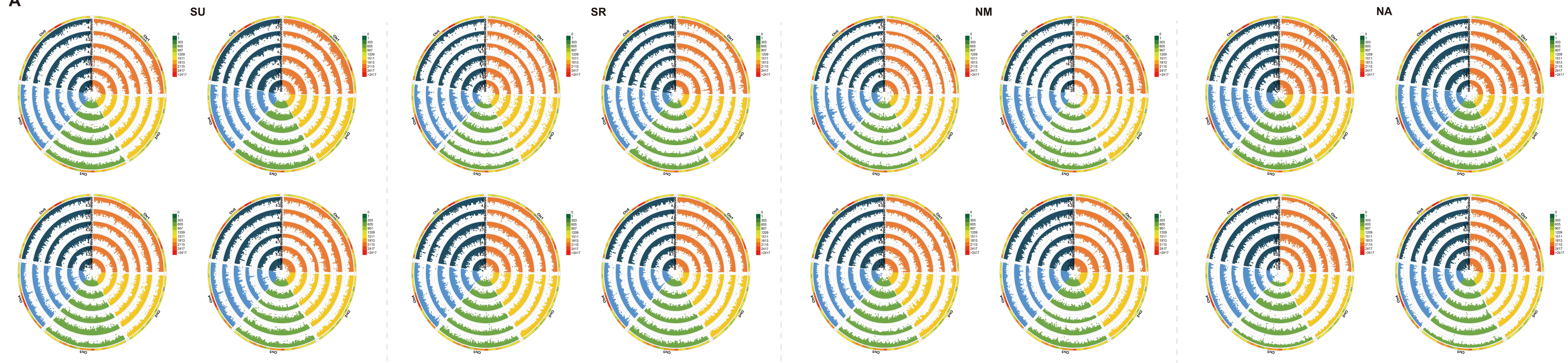

B

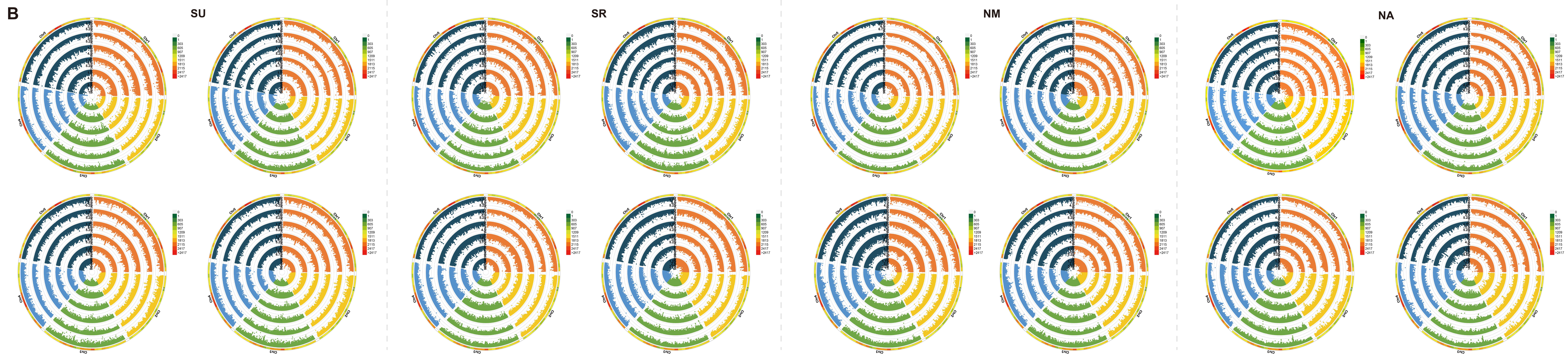

Supplement: Supplementary file 9 [file Image_1.pdf]

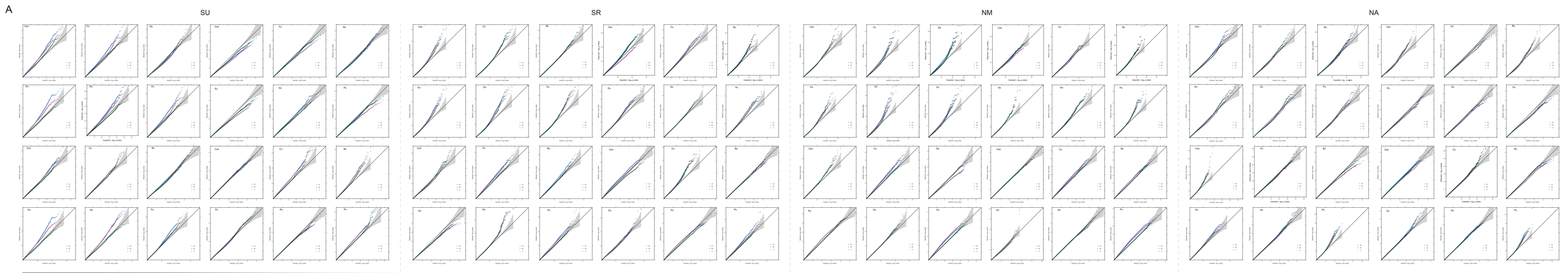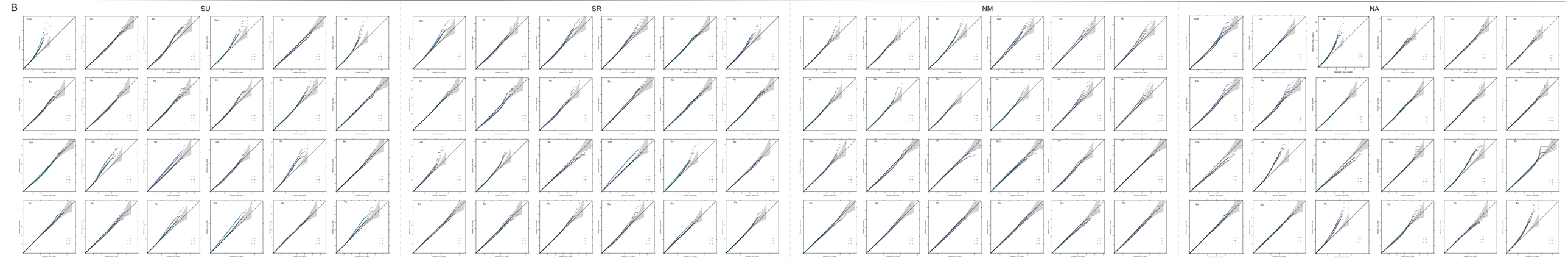

Supplement: Supplementary file 10 [file Image_2.pdf]
